# Oxygen metabolism in oral cancer: HIF and GLUTs (Review)

**DOI:** 10.3892/ol.2013.1371

**Published:** 2013-06-03

**Authors:** KARUZA MARIA ALVES PEREIRA, FILIPE NOBRE CHAVES, THALES SALLES ANGELIM VIANA, FRANCISCO SAMUEL RODRIGUES CARVALHO, FÁBIO WILDSON GURGEL COSTA, ANA PAULA NEGREIROS NUNES ALVES, FABRÍCIO BITU SOUSA

**Affiliations:** 1 Division of Oral Pathology, School of Dentistry, Federal University of Ceará, Sobral Campus, Sobral 62010-560;; 2 Division of Stomatology, Department of Clinical Dentistry, School of Dentistry, Federal University of Ceará, Fortaleza Campus, Fortaleza 60430-160;; 3 Divisions of Stomatology and Oral and Maxillofacial Surgery, Federal University of Ceará, Sobral Campus, Sobral 62010-560;; 4 Divisions of Radiology and Stomatology, School of Dentistry, Federal University of Ceará, Sobral Campus, Sobral 62010-560;; 5 Division of Stomatology, Department of Clinical Dentistry, School of Dentistry, Federal University of Ceará, Fortaleza Campus, Fortaleza 60430-160, Brazil

**Keywords:** cancer, carcinoma, squamous cells, glucose transport proteins, facilitative hypoxia-inducible factor-1α, oral cancer

## Abstract

Oral cancer is a significant cause of morbidity and mortality, and has a poor prognosis. This has encouraged additional studies into factors that may affect the development of this disease. The biological behavior of malignant neoplasms is complex. Studies have investigated the energy metabolism of tumor cells, in an endeavor to elucidate the tumor biology. The identification of molecular signatures and mechanisms, in order to understand tumor progression, may facilitate the identification of novel predictive and prognostic markers. Pathways that influence tumor progression, such as those involving hypoxia-inducible factor (HIF) and glucose transporter (GLUT) proteins, have been the targets of recent studies.

## Contents

1. Introduction
2. Hypoxia-inducible factor (HIF)
3. Glucose transporter (GLUT) proteins
4. Conclusion

## 1. Introduction

Cancer is the focus of scientific studies and investigations into oral pathology, as it is a significant cause of morbidity and mortality, and has a poor prognosis. This has encouraged additional studies that are concerned with factors that may alter the development of this disease. Oral squamous cell carcinoma, or oral epidermoid carcinoma (OEC), is the most common clinical entity among the malignant oral neoplasias, accounting for ∼90% of oral cancer cases (1,2).

OEC presents with heterogeneous clinical, pathological and biological aspects, and its development and progression are promoted by multiple alterations at a cellular and molecular level in the squamous epithelium (3,4). A loss of heterozygocity on specific chromosomes, microsatellite instability and mutations in the tumor suppressor genes, p53 and p16, which are important in cell cycle regulation, are associated with distinct phases of tumor progression (5). In order to predict the potential aggressiveness of OEC, factors in addition to the tumor classification and staging system [the tumor node metastasis (TNM) staging system] ought to be considered. Thus, scientific studies have focused on the involvement of biomarkers in the progression of OEC, by means of various laboratory methods (6,7).

The biological behavior of malignant neoplasias is complex. The growth and dissemination of cancer depend, not only on neoplastic cell proliferation, but also on the normal host tissue responses; a variety of interactions have been observed between tumor cells, the vascular network, the immune system and supportive conjunctive tissue (8). In order to understand the tumor biology, the energy metabolism of tumor cells has been investigated (9).

Establishing molecular signatures and mechanisms, in order to understand tumor progression, may facilitate the identification of novel predictive and prognostic markers, in addition to new therapeutic targets for the treatment of cancer (5). This is due to the fact that the TMN staging system and the histopathological degree of differentiation are insufficient for predicting the prognosis of OEC (10). Thus, pathways that have an influence on tumor progression have been the targets of previous studies, such as those involving HIF and GLUT proteins (8,11).

Solid tumors may present rapid growth, which exceeds the necessary vascularization, and thus the supply of nutrients and oxygen becomes insufficient for the tumor tissue. Under these conditions of hypoxia, a signaling pathway, the HIF-1 pathway, is activated, which acts as a modulator of an adaptive response to the reduction of oxidative stress in the tumor microenvironment (10). In a hypoxic tumor medium, HIF may act on the genes that regulate glycolytic metabolism, promoting an increase in the rate of glucose uptake by the cell through the transcription of GLUTs. This uptake allows the cell to obtain energy by means of glucose metabolism, as a metabolic change from mitochondrial respiration to glycolysis is frequently observed in the neoplastic cell. Therefore, the increase in glucose uptake by the GLUTs is essential, as it facilitates an increased survival time of the neoplastic cell (12).

The aim of the present study was to conduct a literature review of oral squamous cell carcinoma, focusing on the factors that influence the metabolism of oxygen during tumor growth and progression, and emphasizing the roles of HIF and GLUTs in oral carcinogenesis. A search strategy was implemented in PubMed, selecting only English articles in the period from 2002 to 2012, and which used the terms or combinations of the following descriptors: Oral squamous cell carcinoma, HIF, hypoxia-related proteins, GLUT proteins and tumor progression. A total of 36 scientific studies were selected, which involved laboratory investigations, particularly those including immunohistochemistry, uni- and multivariate analyses, and retrospective, comparative and multi-centric studies. These studies were concerned with the role of HIF and GLUTs in the carcinogenesis of oral squamous cells.

## 2. Hypoxia-inducible factor (HIF)

With rapid tumor growth and expansion, the intratumoral regions may present with hypoxia, which is a state of reduction in oxidative stress to levels below the normal limit, and this is commonly observed in diverse malignant tumors. Characteristically, OEC is a locally aggressive neoplasm with rapid progression, and the oxygen concentration is significantly reduced in OEC tumors (13). All cells of the body require oxygen to perform their normal metabolic functions, which include oxidative phosphorylation. The capacity of cancerous cells to adapt to hypoxia, whether transitory or extensive, is essential for tumor survival. It has been suggested that the invasive and metastatic nature of OEC is a consequence of its adaptation to the hypoxic microenvironment (7,14). An important mechanism for adaptation to reduced oxygen concentrations is the regulation of HIF-1. Oxygenation in solid neoplasms depends on the supply of oxygen and its consumption by the tumor cells. Therefore, hypoxia is a common phenomenon in various types of malignant neoplasms, contributing to the progression of cancer and the selection of the more aggressive phenotype (7,15). The high metabolic rates of tumor cells induce intratumoral hypoxia; this hypoxic stress induces the expression of a complex of genes that regulate homeostasis of the oxygen supply. In addition, HIF-1 is the master regulator of the transcription of these genes. Therefore, HIF-1 mediates adaptive responses at cellular and systemic levels for the maintenance of homeostasis, which is the main mechanism whereby tumor cells respond to acidosis and hypoxic stress (12).

**Figure 1.**
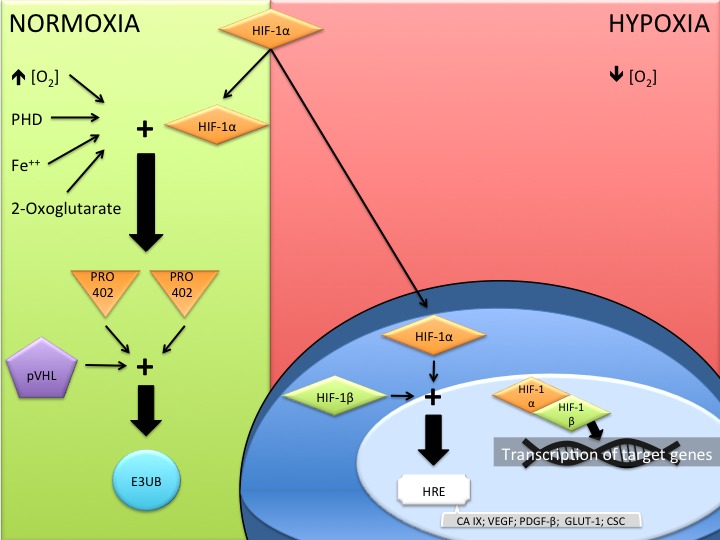
Mechanism of action of hypoxia-induced factor 1 (HIF-1) in normoxic and hypoxic environments. PHD, prolyl hydroxylase domain; PRO 402, proline 402; pVHL, von Hippel-Lindau tumor supressor; E3UB, E3 ubiquitin ligase; HRE, hypoxia response element; CA IX, carbonic anhydrase IX; VEGF, vascular endothelial growth factor; PDGF-β, platelet derived growth factor-β; GLUT 1, glucose transporter 1; CSC, cancer stem cell.

HIF-1 is a basic, helix-loop-helix-PER-ARNT-SIM (PAS) heterodimer composed of α and β subunits. HIF-1α is the oxygen-sensitive subunit that dimerizes with the constitutively expressed β subunit (HIF-1β) (10). HIF-1β, also known as aryl hydrocarbon-receptor nuclease translocator (ARNT), is a 91- to 94-kDa protein. HIF-1α is encoded by a gene located on chromosome 14 (14q21-q24), and is stabilized and accumulated in response to hypoxia. Under the conditions of a normal oxygen supply, HIF-1α is not usually detected or stabilized (7), and is hydroxylated at the proline 402 and 564 (Pro-402 and -564) residues. Following hydroxylation, the HIF-1α residues bond to the von Hippel-Lindau tumor suppressor protein (pVHL) and are rapidly destroyed by an enzyme, E3 ubiquitin ligase (Fig. 1) (16). Another mechanism whereby HIF-1 is inhibited is by the HIF-1 inhibition factor, which hydroxylates asparagine 803 by means of the transactivation domain, and consequently blocks the p300-CREB-binding protein (CBP) coactivator bond (7).

The suppression of tumor suppressor genes, such as p53, phosphatase and tensin homolog (PTEN) and pVHL, may increase the expression of HIF-1α in tumor cells, as well as the action of oncogenes [including ras, SRC and phosphatidylinositide 3 kinase (PI3K) proteins), growth factors (such as the endothelial growth factor) and cytokines (including prostaglandin E2) (12). The role of HIF-1α in carcinogenesis is to signal the message of hypoxia to the cell nucleus, which promotes a response to hypoxic stress. This response involves influencing the control of >100 genes associated with tumor adaptation, such as glycolytic transport, and with alterations in the tumor microenvironment, by stabilizing the pH and by angiogenesis (7). The action of HIF-1α on cancer stem cells (CSCs) has also been suggested, as a high rate of survival, proliferation and expression of these cells has been observed in a hypoxic medium when compared with cells that are in homeostasis (10). In addition, HIF-1α may control the expression of vascular endothelial growth factor (VEGF) and erythropoietin (EPO) genes, resulting in the promotion of tumor angiogenesis, as both stimulate endothelial cell proliferation and migration, and EPO alone promotes an increase in the proliferation and growth of various human neoplasias. Thus, an increase in HIF-1α expression may influence oral cancer progression by promoting tumor angiogenesis and by direct stimulation of tumor cell growth (17,18).

It has been demonstrated that HIF-1α expression has a significant effect on tumor progression. Patients with oral neoplasias with an elevated rate of HIF-1α expression present a 3.49-fold lower survival rate when compared with such patients with limited expression of this protein (7). HIF-1α is involved in tumor carcinogenesis and progression; Koukourakis *et al* (19) observed that overexpression of HIF-1α was correlated with a more aggressive behavior of oral carcinoma and an elevated resistance to radiotherapy treatment. In addition, Lin *et al* (18) observed that an increase in HIF-1α expression was correlated with a poor prognosis for cases of OEC, by univariate analysis.

Invasion and metastasis are key characteristics of malignant neoplasias and represent the primary cause of cancer-related mortality (13). It has been suggested that HIF-1α is correlated with a higher rate of aggressiveness and metastasis, promoting mutations and stimulating angiogenesis by the activation of VEGF, inducing the proliferation, differentiation and migration of vascular endothelial cells by means of the increase in capillary permeability, as well as the reduction in apoptosis (20). Tumor cells interact with extracellular matrix (ECM) proteins by molecular rearrangement on the cell surface, and cell adhesion and integrin molecules promote binding to the ECM. Thus, tumor hypoxia induces an accumulation of HIF-1α, which in turn increases the expression of integrin α5 and fibronectin in the tumor cells, facilitating binding to the ECM that is rich in fibronectin, and providing cell invasion potential at the cell surface (13).

In addition to hypoxia, tumor cells suffer acidosis-induced stress and an increase in interstitial fluid pressure, and present a greater glucose requirement (12). Studies have demonstrated that cancerogenous cells metabolize a substantial quantity of extracellular glucose, and that a subset of cells may utilize glutamine, a free amino acid abundant in muscle tissue that may act as a source of energy (21–28). In general, neoplastic cells in a hypoxic medium require the uptake of glucose, with the aim of increasing energy levels by means of glucose metabolism (to obtain energy in the form of ATP). Therefore, it is necessary for the cells to facilitate the process of glucose uptake from the extracellular medium. Various GLUTs have been observed in malignant tumors, revealing an important role of GLUTs in the maintenance of neoplastic cell survival, and tumor progression and growth (10).

## 3. Glucose transporter (GLUT) proteins

Cancerogenous cells have a high rate of glucose uptake and glycolytic metabolism, and thus, tumor cells exhibit significantly different metabolic activity compared with that involved in normal eukaryotic cell homeostasis. When there is a limited oxygen supply, and it is therefore not possible to obtain energy by mitochondrial respiration, normal eukaryotic cells maximize their energy production by the combination of traditional energy pathways, including glycolysis, the carboxylic acid cycle and the electron transport chain. Thus, the cells efficiently convert the glucose molecule into carbon dioxide and water, maximizing ATP production and potentially reducing NADPH production (29–31). Normal cells obtain only 10% of their energy by glycolysis, with the remainder being the result of mitochondrial respiratory activity. However, tumor cells obtain the majority of their energy by glycolysis, maintaining elevated rates of lactate production, which is sufficient for tumor cell survival in a hypoxic environment. Glycolysis generates a net gain of only two molecules of ATP per glucose molecule, a markedly smaller amount of energy compared with the net gain of 38 molecules of ATP produced by respiration. Thus, neoplastic cells require an increased glucose uptake that is essential to obtain sufficient energy (10).

Glucose is transported into the cell by means of GLUTs, which are present in all type of cells, and have a variable availability in the tissue distribution and a variable affinity for glucose. GLUTs are a family of proteins that mediate glucose transport through the membrane without depending on energy (26,29). At present, various isoforms of GLUT have been described, and the expression of these is cell-specific and subject to extracellular medium control. Functionally, the GLUTs regulate the movement of glucose between the extracellular medium and the intracellular compartments, maintaining the glucose supply available for cell metabolism (32). The GLUT family was originally proposed to comprise 12 members (Table I); however, novel forms of GLUTs have been described, resulting in a total of 14 known GLUTs (33) that have different affinities for glucose and other hexoses, such as fructose (24,25). This family of transmembrane proteins appears to be regulated by proto-oncogenes, which are present in normal cells, and growth factors. The transport stimulation mechanisms include the translocation of GLUTs to the plasma membrane, and the activation of the transporters at the presynaptic terminals in the plasma membrane, which are regulated by a PI3-kinase-dependent signaling pathway (22,25).

The first GLUTs of this family to be identified were GLUT-1 and -3, which are expressed at variable levels in various human tissues, suggesting that these constitutive isoforms may be responsible for basal glucose uptake. GLUT-2 is expressed in the hepatocytes, where it mediates glucose transport through the membrane. GLUT-4 is expressed in tissues in which glucose uptake is regulated by insulin, including adipose tissue, and skeletal and myocardial muscle, by acute insulin stimulation. The high-capacity transporters, such as GLUT-2 and -4, are normally restricted to cells that do not divide. GLUT-5 is expressed in the small intestine and sperm cells, acting as a fructose transporter (22). GLUT-1 is not detected in large proportions in the cells of normal tissues, with the exception of erythrocytes, germinative cells of the testes, renal tubules, the perineurium of the peripheral nerves and endothelial cells of the blood-brain barrier. Its overexpression has been observed in various types of malignant tumors, such as carcinomas of the colon, lung, breast, esophagus, stomach, ovary and biliary vesicle, as well as malignant mesothelioma, and squamous cell carcinoma of the head and neck (25).

It has been demonstrated that solid malignant tumors with a rapid rate of growth and proliferation are characterized by elevated rates of glucose use and uptake, due to the energy demand required for uncontrolled proliferation, based on the increased and/or atypical expression of multiple isoforms of GLUT (10,22,24,25,29). This elevated expression of GLUTs is correlated with a risk of metastasis and a worse prognosis (9,11,32,33).

Studies, such as the study by Weiner *et al* (34), have highlighted the importance of the correlation between the immunoreactivity of GLUTs, including GLUT-1, and clinical and radiographical findings, when the cytological findings are ambiguous. Ayala *et al* (11) performed an immunohistochemical marking of GLUT-1 and -3 in OEC, and observed an elevated expression of GLUT-1 in the majority of cases; whereas GLUT-3 was identified in 21.1% of the studied OEC samples, in accordance with the fact that this protein is not found in the normal oral epithelium. This suggested that GLUT-1 may be used as an indicator of prognosis, due to its high immunoexpression that is likely to be correlated with the increase in glycolytic metabolism in more aggressive neoplastic cells. A study by Kunkel *et al* (35) analyzed the immunoexpression of GLUT-1 in 118 cases of OEC and observed an inversely proportional correlation between the expression of this protein and the survival rate of patients. This suggested that glucose transport acted in a determinant manner for the promotion of the tumor phenotype, and that GLUT-1 may be considered a negative prognostic survival biomarker in patients with OECs. This was concordant with the study by Ayala *et al* (11), in which it was proposed that this protein be used as a potential marker of tumor progression.

Fukuzumi *et al* (22) observed the elevated immunoexpression of GLUT-1 in oral squamous cell carcinoma, and suggested that this protein may be important in the pathogenesis of the disease. The atypical expression of GLUTs in oral squamous cell carcinoma does not necessarily signify an increase in the rate of glucose transport to the tumor cell, in order to promote substrate levels for obtaining the energy that is important for tumor cell survival and growth; it may involve the deregulation of gene expression in specific tissues and cancerogenous cells. Studies have demonstrated that an overexpression of GLUT-1 and -3 was associated with certain solid tumors with greater potential malignancy and lower survival rates (28,29,36). Additionally, Ohba *et al* (25) demonstrated the use of GLUT-1 as an indicator of the depth of invasion with a trend towards a worse prognosis in patients, as a result of an increase in the possibility of nodal metastasis.

The immunoexpression of GLUT-1 and -3 may be associated with the clinical profile of a group of cells in a subset of patients with oral squamous cell carcinoma, who present with a distinctly poor prognosis. The increase in glycolytic metabolism through the action of these proteins in more aggressive tumor cells indicates a potential prognostic value that may enable patients to be stratified with regard to risk (11). Fluorine-18 fluoro-2-deoxy-D-glucose (FDG) is a glucose analog, which, like glucose, is present at high levels in malignant tumors. Positron emission tomography (PET) with FDG has become a novel, noninvasive, imaging diagnostic field. This technique is utilized and favors the initial diagnosis, the evaluation of the extension of the disease and prognosis, and the treatment planning and monitoring, in addition to the detection of recurrences of the disease (28).

Although it remains widely used, the TNM staging system alone is not capable of establishing a true assessment of the patient’s individual prognosis. In order to predict the potential aggressiveness of oral neoplasias, factors additional to the TNM staging system ought to be considered, such as the use of immunohistochemical biomarkers, with the goal of determining whether they exhibit a greater advantage as a prognostic factor (7).

Studies investigating GLUT-1 and HIF-1 proteins identified that these proteins presented with elevated expression in oral OEC, and were correlated with a worse prognosis. This suggests that GLUTs and HIF-1 may be used as markers of tumor prognosis and progression (10).

## 4. Conclusion

Recent studies have encouraged the analysis of HIF-1 and GLUTs in OEC, assessing these proteins as potential prognostic markers. This, in turn, enables the optimization of therapeutic strategies in the treatment of mouth cancer, as these proteins may be associated with the events of carcinogenesis and exert a significant impact on the survival of patients affected by OEC.

## Figures and Tables

**Table I.** The GLUT family.

Author, year	Isoform	Main tissue localization	Transport
Muecker, 1985; Gould, 1991	GLUT-1	Erythrocytes, brain and ubiquitous	Glucose
Fukamoto, 1988; Gould, 1991	GLUT-2	Liver, pancreas, intestine and kidney	Glucose (low affinity) and fructose
Kayano, 1988; Gould, 1991	GLUT-3	Brain	Glucose (high affinity)
Kukamoto, 1989; James, 1989	GLUT-4	Heart, muscle, WAT, BAT and brain	Glucose (high affinity)
Kayano, 1990; Davidson, 1992	GLUT-5	Intestine, testes and kidney	Fructose and glucose (very low affinity)
Doege, 2000; Lisinski, 2001	GLUT-6	Brain, spleen and leucocytes	Glucose
Joost e Thorens, 2001	GLUT-7	ND	ND
Carayannopoulos, 2000; Doege, 2000; Ibberson, 2000; Lisinski, 2001	GLUT-8	Testes, brain and other tissues	Glucose
Phay, 2000	GLUT-9	Liver and kidney	ND
Dawson, 2001; McVie-Wylie, 2001	GLUT-10	Liver and pancreas	Glucose
Doege, 2001; Wu, 2002; Sasaki, 2001	GLUT-11	Heart and muscle	Glucose (low affinity) and fructose
Rogers, 2002	GLUT-12	Heart, prostate, muscle, small intestine,	ND WAT and brain

Adaptation of Wood and Trayhurn (33). GLUT, glucose transporter; WAT, white adipose tissue; BAT, brown adipose tissue; ND, not determined.
